# Maths on microbes: adding microbial ecophysiology to metagenomics

**DOI:** 10.1111/1751-7915.12233

**Published:** 2014-12-09

**Authors:** Wilfred F M Röling

**Affiliations:** Molecular Cell Physiology, Faculty of Earth and Life Sciences, VU University AmsterdamBoelelaan 1085, 1081 HV, Amsterdam, the Netherlands

Microbial ecology has shifted in the last 30 years from a field strongly depending on culturing and microscopy to a discipline dominated by cultivation-independent molecular analysis. Nowadays, metagenomics (for simplicity, I include in metagenomics also high-throughput sequencing of phylogenetic and functional marker genes) allows unprecedented insight into the composition and functional potential of microbial communities, while metatranscriptomics, metaproteomics and metabolomics inform on the expression of functional potential. Yet it is easy to lose sight of the forest for the trees: certainly for engineered systems, like waste water treatments and bioremediation, one is in the end more interested in what all species together do than what their individual identities and activities are.

While enormous amounts of metagenomic data are generated, these data are currently heavily underexplored. First, for many genes we currently do not know what they encode for. More important, the data are generally explored by ‘simple’ multivariate analysis on gene lists. To illustrate this point, take oil-polluted marine sediments in which hydrocarbon oxidation occurs by sulfate reducers and aerobic microorganisms: standard data analysis will give reads of genes belonging to these two different functional groups equal weight even though the sulfate reducers had to degrade about 10 times more hydrocarbon to produce one new cell than aerobic microorganisms. I foresee that by incorporating microbial ecophysiology into metagenomic data analysis, we will gain much better insight into microbial communities and their functioning.

Many of the components to achieve this goal are already available and will be synergetic when combined. A key component is the concept of ‘reverse ecology’: the evolution and ecology of a species are anchored in its genome, and by large-scale comparison of the ecophysiological properties of species to their genomes and genome-derived metabolic networks we can move forward to linking metagenomic data to community functioning (Röling and Van Bodegom, 2014). ‘Reverse ecology’ uses systems biology modelling of metabolic networks, generally of species that have been isolated and characterized in monoculture (Levy and Borenstein, 2012).

Availability of monocultures is an important drawback still to microbial ecology; we only have access to a relatively small proportion of microbial species found in nature, heavily biased to a few phyla. Thus, more emphasis is needed on physiological characterization of not-yet-cultured species. Cultivation-independent single-cell approaches are rapidly advancing, and single prokaryotic cell transcriptomics is coming into view (Kang *et al*., 2011) and will contribute to revealing in situ physiology. Yet, to obtain more extensive physiological information (e.g. growth rates, growth yields, substrate uptake rates and affinities) for use in ‘reverse ecology’, culturing still appears to be the most effective approach for the near future. In fact, cultivation-independent single-cell analysis may also assist here: metabolic network reconstruction on the basis of single-cell genomic data, overlaid with transcriptomic data, will inform how the microorganism of interest connects to its environment, e.g. which nutrients it may need to take up from its environment, and contribute to the design of isolation strategies.

Thus, microbial ecologists need in part to return to the situation before the advent of molecular microbial ecology, and focus more on culturing. However, enrichment and isolation strategies were in the past, and still are, often ill-considered in light of the environmental context of the microorganism of interest. The favourite method was batch cultivation in liquid medium with unrealistic high concentrations of nutrients, which hardly mimics the environment most microorganisms live in: microbes tend to grow attached, at very low growth rates with low concentrations of nutrients. Nowadays, high-throughput micro-culturing under environmental relevant conditions is possible. An approach like the micro-Petri dish (Ingham *et al*., 2007) can be combined with gradients in nutrients and slow diffusion of substrates, enabling slow growth after dilution-to-extinction of inocula, and allowing for subsequent screening of micro-cultures by microscopy and (meta)genomics.

Culturing was in the past primarily aimed at obtaining pure isolates, capable of performing a particular activity of interest, e.g. hydrocarbon degradation. While obtaining pure isolates is still important and rewarding, I am expecting that more scientific progress will be achieved by studying the enrichments from which isolates typically were acquired. The most illustrative example that comes to mind is the detoxification of chlorinated ethenes by *Dehalococcoides* species. These slow-growing microorganisms are hard to isolate and are often propagated in enrichments, where they grow to higher cell densities than in pure cultures. *Dehalococcoides* strongly dependent on the activities of other species, not only do they need to be supplied with hydrogen by fermenting microorganisms, but they also require other microorganisms for vitamin provision (Schipp *et al*., 2013) and produce carbon monoxide that is toxic to themselves and needs to be removed by other anaerobes (Zhuang *et al*., 2014). Thus, the importance of the activities of microorganisms that indirectly contribute to important processes and interact with the microorganisms that actually perform the activity of interest can be revealed by studying enrichments. These enrichments have generally low diversity, allowing for in-depth metagenomic analysis to retrieve complete genomes, and linking these genomes to each other and to the physiology of the enrichment. The insight obtained from these analyses should help make better sense of environmental metagenomic data.

When studying microbial physiology, it is again important to avoid the pitfalls of the past, when the conditions for physiological characterization were largely similar to those employed for isolation: high substrate concentrations as pure culture, etc. In real life, species undergo short periods of very fast growth and long-term very slow growth. The retentostat, a continuous culture device with biomass retention, allows for studying very slow growth at the molecular level (Marozava *et al*., 2014). Also, the micro-Petri dish and other micro-culturing approaches again will be very valuable: these tools can be used to mimic dynamic and fluctuating conditions by imposing gradients and rapid shifts in environmental conditions. Enrichments, isolates and artificial consortia, assembled from two or more isolates or enrichments, can be studied at high throughput, in combination with metagenomic analysis. I expect that by high-throughput studying of microbial consortia, the function of many currently uncharacterized genes will also be deciphered, which will further aid the interpretation of environmental metagenomic data to reveal microbial community functioning.

## Conflict of interest

None declared.
